# A Depth-Based Head-Mounted Visual Display to Aid Navigation in Partially Sighted Individuals

**DOI:** 10.1371/journal.pone.0067695

**Published:** 2013-07-03

**Authors:** Stephen L. Hicks, Iain Wilson, Louwai Muhammed, John Worsfold, Susan M. Downes, Christopher Kennard

**Affiliations:** 1 The Nuffield Department of Clinical Neurosciences, University of Oxford, Oxford, United Kingdom; 2 The Royal National Institute for Blind People, London, United Kingdom; 3 Oxford University Hospitals NHS Trust, Oxford, United Kingdom; 4 Nuffield Laboratory of Ophthalmology, University of Oxford, Oxford, United Kingdom; 5 NIHR Biomedical Research Centre, Oxford, United Kingdom; ICREA-University of Barcelona, Spain

## Abstract

Independent navigation for blind individuals can be extremely difficult due to the inability to recognise and avoid obstacles. Assistive techniques such as white canes, guide dogs, and sensory substitution provide a degree of situational awareness by relying on touch or hearing but as yet there are no techniques that attempt to make use of any residual vision that the individual is likely to retain. Residual vision can restricted to the awareness of the orientation of a light source, and hence any information presented on a wearable display would have to limited and unambiguous. For improved situational awareness, i.e. for the detection of obstacles, displaying the size and position of nearby objects, rather than including finer surface details may be sufficient. To test whether a depth-based display could be used to navigate a small obstacle course, we built a real-time head-mounted display with a depth camera and software to detect the distance to nearby objects. Distance was represented as brightness on a low-resolution display positioned close to the eyes without the benefit focussing optics. A set of sighted participants were monitored as they learned to use this display to navigate the course. All were able to do so, and time and velocity rapidly improved with practise with no increase in the number of collisions. In a second experiment a cohort of severely sight-impaired individuals of varying aetiologies performed a search task using a similar low-resolution head-mounted display. The majority of participants were able to use the display to respond to objects in their central and peripheral fields at a similar rate to sighted controls. We conclude that the skill to use a depth-based display for obstacle avoidance can be rapidly acquired and the simplified nature of the display may appropriate for the development of an aid for sight-impaired individuals.

## Introduction

There are approximately 285 million people worldwide living with a visual impairment and almost 39 million are considered blind [1]. The leading causes of visual impairments in developing countries are cataracts and uncorrected refractive errors, while in developed countries age-related macular degeneration (AMD), diabetic retinopathy and glaucoma are most prevalent [2]. In the UK the annual health care cost for hospitalisation as a result of visual impairment is in excess of £250 million annually [3].

Contrary to popular belief, “blind” individuals do not usually lose all perception of light. Total blindness is relatively rare and the majority retain some residual vision which, while too low to perform sight-based tasks, is often sufficient to detect the location, motion and brightness of light. Electronic visual aids have been devised to support low vision by increasing the contrast, size, edge visibility or semantic density of a live video image. Such techniques have been shown to improve object recognition, letter reading, face detection and can also assist with navigation [4], [5], [6], [7], [8]. In conditions such as tunnel vision or retinitis pigmentosa where the central visual field remains relatively intact, individuals can make use of augmented high resolution central imagery for navigation. For example Peli et al (1991, 2009) [6], [8] have developed a technique whereby live video acquired from a head-mounted camera is first processed to enhance edges and then reduced in size to fit within a person's remaining visual field. This essentially increases the wearer's field of view while also enhancing the perception of object boundaries and features. However, individuals with broader field sight-loss including macular degeneration, diabetic retinopathy and cataracts are unlikely to benefit from such approaches due to difficulties in perceiving focused light.

Little research has been done on non-invasive visual augmentations for severely sight-impaired or “legally blind” individuals. Due to sight limitations inherent in blindness, auditory or haptic sensory stimulation are often the focus of wearable aids for this group. Sensory substitution involves the use of tones or vibrations to convey the characteristics and position of nearby objects and obstacles [9], [10], [11], [12], [13], [14], [15]. Auditory sensory substitution in particular has been well studied. Results of a recent study of congenitally blind participants reported that the use of sound to describe a visual form elicits brain activity in areas commonly associated with visual recognition [16]. While auditory sensory substitution is developing as a potential aid, its widespread adoption may be limited due to the inherent difficulty of translating auditory information into visual spatial awareness, as well as long training times and the potential masking of important ambient environmental sounds. For the demands of real-time navigation, a sight impaired individual might benefit from a form of *visual* augmentation that conveys the size of object and their spatial arrangement in a simple and high contrast manner.

The development of a wearable visual aid for people with no more than residual vision requires two main elements. Firstly, an appropriate display apparatus, and secondly the development of imaging techniques that produce unambiguous and timely spatial information.

### Appropriate Eyewear

The severe limitations of vision in legal blindness presents a significant challenge to the design of a wearable display. Off-the-shelf video headsets, such as those used in virtual reality applications, aim to produce the effect of a large screen at a removed distance. Such displays are limited in their field of view (typically 30–40 degrees horizontal) and require the wearer to have a normal range of visual acuity. The requirements for a navigational aid are quite different. Situational awareness and obstacle avoidance benefit from a wide field of view (the typical human binocular field of view is over 120 degrees in horizontal [17]). Such a wide field can be achieved by positioning electronic displays close to the eyes, however without optics to focus the light, the resulting image will be blurry. Considering the poor vision of the intended wearer, image sharpness may not be a major concern. As a result of the blurring, a much lower resolution display in the order of hundreds of pixels per eye (as opposed to hundreds of thousands in consumer products), can be produced and may provide enough detail for simple obstacle awareness.

### Computer Vision

**Figure 1:**
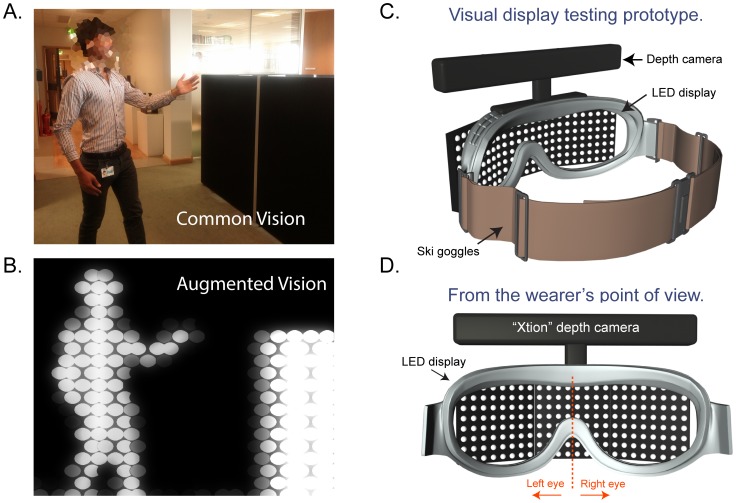
Depth-to-brightness imaging and prototype headset. The role of the software was to transform a depth map into a viewable image by converting distance into brightness, such that closer images appear brighter (A. and B.). In the example above two large forms easily identifiable as a person and a small partitioned wall. Increasing distances are represented as a gradual darkening towards a pre-set depth limit. The prototype visual display designed for use in this study consisted of a depth camera and a horizontal array of LEDs mounted on ski goggles (C. and D.). The display was split binocularly with 12×8 LEDs per eye. The individual in this figure has given written informed consent, as outlined in the PLOS consent form, to publication of their photograph.

**Figure 2:**
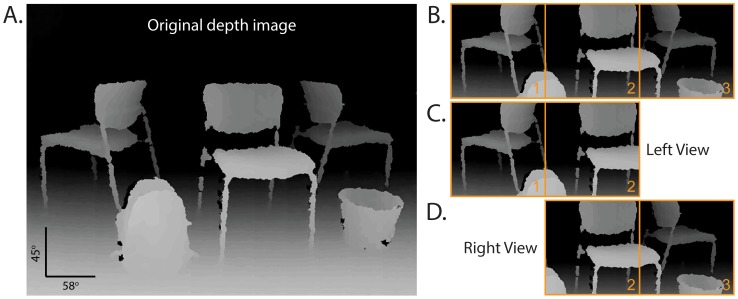
Distributing a depth image across the binocular display. (A.) A single frame from the depth camera showing several chairs and obstacles. In order to generate a correctly proportioned display, only the central strip was presented (B.). This was divided into two binocular viewpoints (C. and D.) where the central portion (2) was shared between both eyes and unique thirds (1 and 3) were presented in the periphery of each display.

A major requirement for a navigational aid is the ability to locate nearby objects rapidly and accurately to allow obstacle avoidance at walking speed. Computer vision techniques have been developed that can highlight particular classes of objects such as floors, walls, people and cars [18], [19], however the computational demands for these algorithms are usually too high for live, high frame-rate displays using present day portable computers. Depth cameras developed for consumer electronics (eg. Kinect by Microsoft) offer an efficient way to quickly identify individual objects in a scene while consuming minimal computational resources. This technology works by projecting a field of infrared points onto nearby surfaces, capturing the field with an infrared camera and then analysing the displacement of points to construct a depth map. The resultant two-dimensional depth map accurately preserves the shape and position of nearby objects (see Figures 1 and 2).

A depth map can be translated into a useful visual image by applying filters that mimic our own intuitive sense of distance. Perceptually, object surface properties such as contrast and luminance provide reliable information on the relative distance of an object [20]. Retinal image size and the level of surface detail are also important cues to distance [21]. We reasoned that a similar approach could be applied to a depth map by representing distance as brightness, such that nearby objects appear large and bright, and dark or black regions inform the wearer of distant objects or safe unobstructed paths.

To assess the feasibility of a depth-based visual display, we developed a wearable display encompassing a low-resolution LED display (96 pixels per eye), a depth camera and a portable computer. The displays did not carry any focusing optics and were mounted too close to the eyes to form a sharp picture on the retina. The main aim of the displays was to provide simplified spatial awareness for nearby object detection. We performed two experiments to test this design:

Study 1 examined whether sighted volunteers could learn to use a depth-to-brightness display to navigate a set of obstacle courses. Sighted volunteers were chosen for this part of the study to allow us to monitor learning rates of a complex and dynamic visual stimuli without the complication of different visual fields, contrast sensitivities and levels of acuity that would be expected in a population of severely sight impaired individuals.

Study 2 was designed to see if blind and partially-sighted people were able to perform a visual search task using static cues on a similar low-resolution and unfocussed display.

## Methods

**Figure 3:**
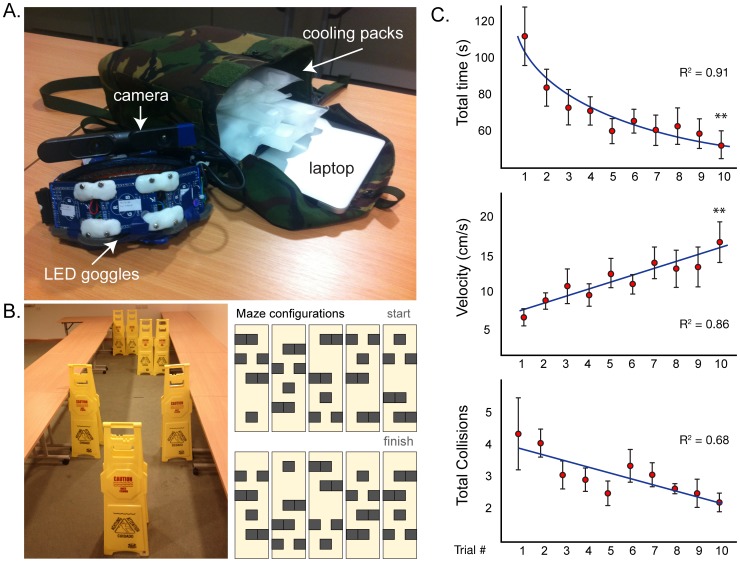
Obstacle avoidance task. (A.) The visual display in a portable format including: depth camera, goggle mounted LEDs, laptop and cooling packs. (B.) A photo of one configuration of the obstacle course and top-down schematics of all ten configurations. (C.) Graphs showing the three main outcome measures averaged across all subjects for each of the ten randomly assigned courses. The total time to completion shows a logarithmic decrease from an average of 112 seconds for the Trial 1 down to 52 seconds by Trial 10 (R^2^ = 0.91). Median velocity increased linearly across trials from 17 to 31 cm/s (R^2^ = 0.86). The average number of collisions decreased linearly across trials from 3.9 at Trial 1 to 1.7 at Trial 10 (R^2^ = 0.68). Bars represent one standard error.

The testing device was a head mounted display made up of 24×8 colour LEDs (light emitting diodes) comprised of three 60 mm^2^ LED matrices (maximum intensity 40 millicandella/LED, Colorduino, ITead Studio) arranged horizontally in a slight arc and attached to the front of a pair of ski goggles (Figure 1). The horizontal field of view was approximately 120°. A semi-transparent film was inserted in front of the LEDs to diffuse the individual points of light. An infra-red depth camera (Primesense 1080, ASUS Xtion) with a field of view of 58°H and 45°V was mounted on the bridge of the goggles. The LEDs and camera were connected to a portable computer (2 Ghz Core2Duo) running software to acquire a depth map and output the result to the LED displays. The display frame rate was between 25–30 frames per second and system performance was monitored remotely via a wireless link to a tablet computer. While completing the obstacle courses participants carried the computer in a small backpack. Phase-change cooling packs were inserted alongside the computer to maintain an optimal operating temperature (see Figure 3).

The LED array was divided in half to provide two 12×8 pixel displays for each eye. The nasal halves of each eye’s display (a 6×8 portion) showed the central third of the depth image. The temporal halves of each display showed the peripheral third of the depth image that was unique to each eye (Figure 2). The combination of the left and right eye views produced a total screen resolution of 18×8 pixels (aspect ratio 2.25∶1) however the camera's aspect ratio was 1.3∶1. Therefore to maintain a true aspect ratio only a central horizontal strip from the camera, 58°H and 26°V, was presented on the LED displays (Figure 2). Minor adjustments to the degree of separation of the displays were applied based on the individual’s inter-ocular distance.

The maximum and minimum distances detected reliably by the camera were 8 and 0.5 metres respectively. Software converted the distance to objects into a 12 point linear brightness scale where maximum brightness was applied to surfaces at 0.5 m and a brightness value of zero for surfaces 8 m and beyond. To help increase contrast between foreground and background surfaces, an algorithm was employed which monitored the ratio of light to dark on the display and maintained a 50∶50 ratio by continuously altering the distance to brightness scale. For example, when looking around a large room, the user may be able to see objects several meters away, however if they raised their own hand in front of the camera a large portion of the display would be filled causing the algorithm to reduce the maximum visible distance and thus maintaining a constant ratio of light to dark.

All clinical investigation were conducted according to the principles expressed in the Declaration of Helsinki. Written informed consent was obtained from participants before the study commenced. The consent forms were countersigned by the lead experimenter (SH) and the documents were filed at the Nuffield Department of Clinical Neurosciences in the University of Oxford. The experimental protocol, method of recruitment, information sheet and consent procedure were approved by the local ethics committee at the University of Oxford in the case of Study 1, and by South Oxford REC in the case of Study 2.

### Study 1. Navigation and Obstacle Avoidance

Seven healthy controls, ages 22 to 36 were recruited from the staff population at the John Radcliffe Hospital. All had normal, or corrected to normal vision. The study had two main aims: Firstly to determine whether it was possible to use the apparatus to avoid obstacles in a small maze, and secondly to quantify the rate at which people were able to learn to use the novel display. Normally sighted rather than sight impaired individuals were recruited for this task in order to have a homogeneous level of visual acuity. All participants were naïve to the apparatus prior to testing. Participants were instructed on how the apparatus functioned and allowed 2–3 minutes to become used to the depth-to-brightness display. Otherwise, there was no specific training prior to testing.

Participants were asked to traverse a narrow path without colliding with the maze boundaries or any obstacles. The maze consisted of a narrow corridor (6×2 metres) with five obstacle points. One of five different obstacles (gate, bifurcation, left corridor, right corridor, empty space) was arranged at each point. A set of identical ‘wet floor’ signs were used to create the obstacles (Figure 3). Ten different mazes were generated by rearranging the position of each obstacle and participants completed each of the mazes in a random order. Two further depth cameras (ASUS Xtion) using person tracking software (NITE, OpenNI.org) were used to record the location of the participants. Total time taken to complete the maze, median velocity and the number of collisions were recorded.

#### Results

All participants were able to complete the obstacle courses while wearing the visual aid. The average time taken to complete the course decreased in a logarithmic fashion (R^2^ = 0.91) over ten trials from an average of 112 seconds at Trial 1 to 52 seconds at Trial 10. The average median velocity increased linearly (R^2^ = 0.86) from 17 to 31 cm/s from Trial 1–10 and the average number of collisions decreased linearly (R^2^ = 0.68) from an average of 3.9 per course for Trial 1 to 1.7 per course for Trial 10 (Figure 3). A one way repeated measure ANOVA was performed on each of the variables across trials. There was a main effect for Total Time ((F9,54) = 7.29, p<0.00001), and Velocity (F(9,45) = 6.63, p<0.00001), and a marginal but non-significant effect for Total Collisions ((F(9,54) = 1.94, p = 0.06). Planned comparisons were performed to compare trial 1 and trial 10 for each of the variables. Total Time decreased significantly (F(1,6) = 13.8, p<0.01) and Velocity increased significantly (F(1,6) = 18.5, p<0.01) across trials. There was no significant difference in the Total Collisions (F(1,6) = 2.6, p = 0.16).

### Study 2. Augmented Vision in Severely Sight-impaired Individuals

Eighteen volunteers, aged from 20 to 90 years, healthy except for a significant sight impairment were recruited from the general population. Fourteen self-reported as blind and the remaining four were partially-sighted. Conditions included retinal dystrophies (including retinitis pigmentosa), macular degeneration, and smaller sets including glaucoma, Leber optic neuropathy and Stargardt disease (see Table 1).

**Table 1:** Characteristics of sight impaired participants in order of their combined DLTV (Daily Living Tasks Dependent on Vision [22], [23]) score.

Age & Gender	Cause of visual impairment	DLTV score	Blind or partially sighted	Useful residual vision
37 F	Retinitis pigmentosa	6	Blind	No
35 M	Retinitis pigmentosa	8	Blind	No
54 F	Leber optic neuropathy	11	Blind	No
60 F	Macular degeneration	12	Blind	Yes
70 M	Retinitis pigmentosa	13	Blind	No
44 F	Stargaardt disease	20	Blind	Yes
40 F	Glaucoma	22	Blind	No
61 M	Retinitis pigmentosa	23	Blind	No
42 M	Retinal dystrophy	24	Blind	No
28 F	Glaucoma	24	Blind	Yes
90 M	Macular degeneration	25	Blind	Yes
58 F	Sarcoidosis uveitis	28	Blind	Yes
69 F	Retinal dystrophy	28	Blind	Yes
39 M	Retinal dystrophy	33	Blind	Yes
53 M	Retinitis pigmentosa	44	Partially sighted	Yes
89 F	Macular degeneration	45	Partially sighted	Yes
29 F	Hemianopia	55	Partially sighted	Yes
71 F	Cataract	55	Partially sighted	Yes

Participants self-reported whether they considered themselves blind or partially sighted and with or without useful residual vision.

Each participant performed three tasks: a questionnaire dealing with difficulties living with sight-loss, a search task using a head-mounted display, and an ad lib exploration of the prototype display. Due to the proof-of-principle aims of the study detailed information about participants' visual acuities, visual fields and levels of contrast sensitivity was not collected.

#### Daily Living Tasks Dependent on Vision (DLTV)

To acquire general data on the degree to which visual impairment had affected each participant's day-to-day lives, we administered 24 questions from the DLTV [22], [23] a validated scale of sight loss. For each item on the questionnaire the participants were asked to rate, on a 4 point scale, how difficult they found certain daily tasks. The maximum possible score on the DLTV was 72, where 71 or below indicates a level of impaired sight. Additional personal and demographic questions were asked which included a self-report of whether the participant considered themselves (a) partially sighted, (b) blind with useful residual vision or (c) blind without useful residual vision (see Table 1).

#### Head-mounted search task

**Figure 4:**
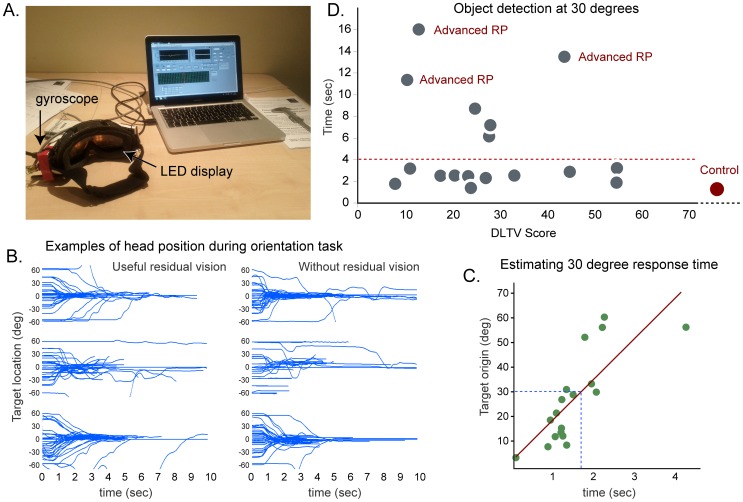
Head-mounted search task with sight impaired individuals. (A.) A pair of ski goggles carrying an array of LEDs and digital gyroscope (IMU-3000, Sparkfun) connected to a computer. An example of the stimuli (a binocular set of illuminated 2×2 squares) is visible on the display. (B.) Six representative sets of head position data from blind individuals orienting towards stimuli appearing between ±60 degrees. As the participant turns to face each new target, the blue head position trace approaches zero. Several undetected targets are apparent at high eccentricities. (C.) This graph from a sight impaired participant shows the linear relationship between target eccentricity and the time to orient. Fitting a line through these points allows a predicted time for a target appearing at 30° to be estimated. (D.) Response times from all participants and a sighted control (in red) to orient towards a target at 30° vs total DLTV score. The majority of those tested performed similarly to the control. Conspicuous outliers above 10 seconds were found in three participants who had extremely constricted fields of view due to retinitis pigmentosa (RP).

To quantify the ability of sight impaired people to see and orientate towards objects in the display, small squares of light (2×2 LEDs) were presented on the goggles at locations between +60° and −60° of the vertical mid line. Participants were asked to physically turn their heads to face any region of light, which could appear anywhere in the visual field. A small digital gyroscope (IMU-3000, Sparkfun) mounted on the goggles tracked the orientation of head and was used to change the position of the stimulus in relation to head movement. The effect of this was to make each stimuli appear stationary in space. Software (LabVIEW, National Instruments) was written to control the stimuli locations and log the gyroscope (at 100 Hz) to provide data on the response rates of the participants. The response rate was quantified as the time taken to move the light from its starting location to zero (i.e. the mid-line). Each participant completed approximately 30 trials at starting positions between −60 and +60 degrees, using light levels they found comfortable (Figure 4).

#### Results

Participants took between 1 and 20 seconds to detect and orient to visual stimuli presented at different positions in their visual fields. As can be seen in the representative traces of Figure 4B, participants took between 1 and 2 seconds to detect the targets before bringing them towards the mid-line (identifiable as the gyroscope position data reaching zero). The gyroscope data for targets presented at zero rarely deviated, suggesting that these targets were detected almost immediately, even in cases of macular degeneration. All participants were able to orient towards targets presented up to ±30° from the midline, however targets at greater eccentricities were increasingly missed by participants with advanced peripheral deficits, such as retinitis pigmentosa.

The time taken to respond to a target increased linearly with the eccentricity of the stimuli's starting position (Figure 4C). By fitting a line through a population of responses we can predict each participant's performance for a standard distance, in this case 30 degrees, which can then be used to compare performance across all participants and against DLTV scores. The time to orient to a target appearing at 30 degrees is shown for all participants (figure 4D), and for reference, a sighted control (whose response time was approximately two seconds). The majority of sight-impaired individuals could orient to targets in approximately the same period of time as a sighted control irrespective of their DLTV score. There are three significant outliers (labelled with red). All three participants had advanced retinitis pigmentosa; two of whom considered themselves completely blind, one with a retinal implant (switched off).

## Discussion

A low-resolution visual display showing the distance to nearby objects as a scale of brightness, and positioned close to the eyes without focussing optics, was sufficient to allow sighted participants to navigate a small obstacle course. All participants could use depth-based information to locate and avoid obstacles, and overall task performance appeared to improve at a logarithmic rate. Walking speed was shown to increase across trials without an increase in the number of collisions, suggesting that participants were rapidly adapting to the novel visuo-spatial display. Logarithmic adaptation rates are seen in visuo-motor tasks where participants learn to use an altered visual display, such as prismatic lenses, to walk around obstacles or manually reach for targets [24], [25]. Such rates are indicative of neural plasticity underpinning learning and adaptation. The high learning rates presented here help to show that depth-based imaging is capable of generating an intuitive visual field, which would be an advantage for an assistive device developed for people advanced in age and unfamiliar with such technology.

Situational awareness generally improves with a wide field of view, and while the LED displays in this experiment provided approximately 120 degrees of horizontal vision, the input image from the camera was restricted to under 60 degrees. In order to present a visible image on the display that was in proportion to the size and scale of objects in the real world, the vertical angle had to be limited to 26 degrees. Participants responded to this reduced vertical field of view by adopting a behaviour of walking with their heads angled downwards to better see the obstacles. A consequence of the limited visual field was that a high proportion of collisions were made with obstacles that they appeared to have passed were still by their side. Further iterations of the apparatus would clearly benefit from a wider horizontal and vertical input image.

Head mounted displays can often cause eye strain as they force the visual system to focus at a fixed depth. The displays in these experiments were unfocussable and when asked participants did not report any discomfort despite wearing the goggles continuously for up to thirty minutes. The distance between the eye and the display in this study was under 3 cm, which is too close to trigger accommodation, thus participants appeared not to attempt to focus on the display but rather looked *through* it. This behaviour was encouraged by creating a binocular display that allowed the eyes to fuse the image at a comfortable distance. However, even through this blurred view, it was easy for the participants of this study to identify the boundaries of objects and their approximate size and position.

Using a similar low-resolution and near positioned display, blind and partially-sighted individuals were able to see and respond to illuminated objects presented throughout their residual visual fields. All sight-impaired individuals were able to perform the head-mounted search task and many of the participants who self-reported that they had no useful residual vision could still complete the task. Predictably, participants with advanced RP (with peripheral vision deficits) were unable to reliably respond to objects presented in the periphery. Conversely, participants with AMD (characterised by a central vision deficit) appeared to have no specific problem detecting targets in any region of their visual fields, including the centre. The close proximity of the display to the eyes did not prevent the participants from finding the targets, nor did it produce symptoms of eye strain over the period of testing.

Having established that healthy controls can use a dynamic low-resolution depth-based display for navigation, and that severely sight-impaired people are able to perform a search task using static visual cues on a similar display, the next stage is to establish whether this technique can improve obstacle avoidance in blind or partially-sighted individuals. Severely sight-impaired individuals are often highly skilled at detecting and tracking object positions by using shadows, reflections and memory. People with macular degeneration often have sufficient peripheral vision in good light to detect obstacles while walking. In order to make a proper estimate of any benefit that this type of display may add, it would be necessary to combine depth-based information with their remaining vision using, for instance, a transparent see-through LED display.

In unquantified discussions with sight impaired individuals using the depth-based version of display, many were able to quickly and accurately detect people at a distance of up to 4 metres away. Furthermore, within ten minutes almost all were able to recognise nearby objects such as walls, chairs and their own limbs. This suggests that depth-based imaging may be an easy to learn and highly intuitive form of visual augmentation. For a more detailed assessment of the usefulness of such a wearable display for different severities of sight loss, the classification of visual acuity and visual fields and contrast sensitivity needs to be performed. For instance, contrast sensitivity is often much lower in visual impairments and some individuals may not be able to detect a scale of brightness indicating depth, rather they may see just a binary image (eg. object/no object).

This study provides evidence of the potential to develop a wearable display that can deliver simplified, rapid and usable object detection for the purpose of obstacle avoidance in people with very limited sight. With refinements to the field of view, screen resolution and portability, such a device may be able to provide intuitive visual enhancements through one's residual vision.
